# Editorial Notes

**Published:** 1883-03

**Authors:** 


### EDITORIAL NOTES.

OUR editorial confrere, Dr. Davidson, with a view to enjoy a long-coveted rest from work, and to extend his field of professional observation, sailed Feb. 24th for London, where he intends to avail himself of the extensive clinical opportunities of that great medical centre. We are promised occasional contributions from his pen, which will be sure to interest our readers and especially those who know from personal acquaintance the ability and scientific attainments of our accomplished collaborator. We wish a hearty *bon voyage* to our absent fellow-worker, and a safe return to a field of professional labor, only awaiting to be cultivated by such an earnest and energetic worker as our friend has proven to be.

